# Layer dependent neural modulation of a realistic layered-microcircuit model in visual cortex based on bottom-up and top-down signals

**DOI:** 10.1186/1471-2202-12-S1-P114

**Published:** 2011-07-18

**Authors:** Nobuhiko Wagatsuma, Tobias C Potjans, Markus Diesmann, Tomoki Fukai

**Affiliations:** 1 RIKEN Brain Science Institute, 2-1 Hirosawa, Wako, Saitama 351-0198, Japan; 2 Japan Society for the Promotion of Science, Japan; 3 Institute of Neuroscience and Medicine, Computational and Systems Neuroscience (INM-6), Research Center Juelich, Juelich, Germany; 4 Brain and Neural Systems Team, RIKEN Computational Science Research Program, 2-1 Hirosawa, Wako, Saitama 351-0198, Japan; 5 Faculty of Biology III, Albert-Ludwigs-University Freiburg, Schaenzlestrasse 1, 79104 Freiburg, Germany; 6 CREST, JST, Hon-machi, 4-1-8, Kawaguchi, Saitama, Japan

## 

**Figure 1:**
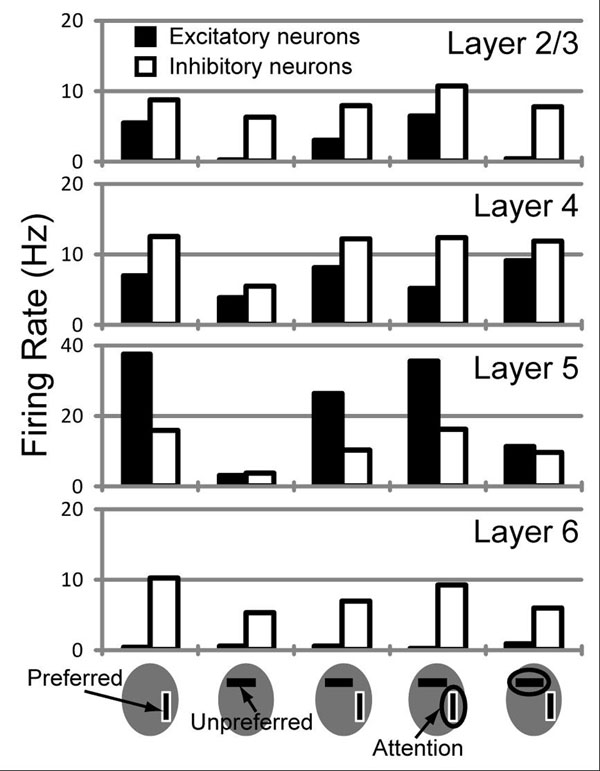
The population firing rates of excitatory (filled bars) and inhibitory (empty bars) neurons are shown for each layer of a column for various combinations of visual and attentional inputs. The preferred stimulus of the column is bordered white. An attended stimulus is circled.

Visual attention allocates the information processing resources of the brain to selected sensory inputs to reduce the processing load. Here, we carried out a large-scale simulation with a layered-microcircuit model of the visual cortex based on current knowledge of cortical neurobiology to explore the complex interaction between bottom-up visual input and top-down attentional signals. The microcircuit model corresponds to two columns of visual cortex. Each column consists of about 40,000 integrate-and-fire neurons comprising layers 2/3, 4, 5 and 6 [1]. These columns interact via lateral inhibition from layer 2/3 excitatory neurons in one column to layer 2/3 inhibitory neurons in the other, so that the full network comprises in total around 80,000 neurons and 300 million synapses. Excitatory and inhibitory neurons in layer 4 of the model receive visual inputs mimicking vertical and horizontal bars. Top-down attention was projected to layers 2/3 and 5 to facilitate the visual processing. In order to investigate the mechanism of visual processing and the attentional effect in the layered-microcircuit model, we compared the response of the model to physiological results for neural and attentional modulation [2]. Our model successfully reproduced previous experimental observations of stimulus presentation and attentional effects on visual neuronal responses in layers 2/3 and 5 (Figure 1). Moreover, the model predicts contrasting differences in the effects of attention between cortical layers: attention to a preferred stimulus of a column enhances neuronal responses of layers 2/3 and 5, the output station of cortical microcircuits, whereas attention suppresses neuronal activities of layer 4, the input station of cortical microcircuits. Further simulations with our model suggest that the specific attentional modulation pattern of layer 4 activity emerges from inter-laminar synaptic connections within the cortical microcircuit and is crucial for the microcircuit to rapidly shift attention to a stimulus different from the one currently attended to. Our multicolumnar model is a canonical microcircuit model for the dynamic selection of multiple sensory inputs to the visual cortices, and makes several testable predictions about the layer-dependence of the response modulations.
